# A Systematic Review of Socioeconomic Indicators and Dental Caries in Adults

**DOI:** 10.3390/ijerph9103540

**Published:** 2012-10-10

**Authors:** Simone M. Costa, Carolina C. Martins, Maria de Lourdes C. Bonfim, Lívia G. Zina, Saul M. Paiva, Isabela A. Pordeus, Mauro H. N. G. Abreu

**Affiliations:** 1 School of Dentistry, Universidade Federal de Minas Gerais, Belo Horizonte 31270-901, Brazil; Email: smelocosta@gmail.com (S.M.C.); carolcm10@hotmail.com (C.C.M.); malu_cb2000@yahoo.com.br (M.L.C.B.); smpaiva@uol.com.br (S.M.P.); isabelapordeus@ufmg.br (I.A.P.); 2 Minas Gerais State Public Health School, Avenida Augusto de Lima, 2061-Barro Preto, Belo Horizonte MG 30190-002, Brazil; Email: liviazina@yahoo.com.br

**Keywords:** epidemiology, caries, adults

## Abstract

Increasing evidence suggests that socioeconomic factors may be associated with an increased risk of dental caries. To provide better evidence of the association between dental caries in adults and socioeconomic indicators, we evaluated the relation between these two conditions in a thorough review of the literature. Seven databases were systematically searched: Pubmed, Cochrane, Web of Science, Bireme, Controlled Trials, Clinical Trials and the National Institute for Health and Clinical Excellence. No restrictions were placed on the language or year of publication. The search yielded 41 studies for systematic review. Two independent reviewers screened the studies for inclusion, extracted data and evaluated quality using the Newcastle-Ottawa scale. The following socioeconomic indicators were found: educational level, income, occupation, socio-economic status and the community index. These indicators were significantly associated with a greater occurrence of dental caries: the subject’s education, subject’s income, subject’s occupation and the Gini coefficient. A high degree of heterogeneity was found among the methods. Quality varied across studies. The criteria employed for socioeconomic indicators and dental caries should be standardized in future studies. The scientific evidence reveals that educational level, income, occupation and the Gini coefficient are associated with dental caries.

## 1. Introduction

There has been a reduction in the prevalence of dental caries in both developed and developing countries [1]. However, the prevalence remains high among populations of low socioeconomic status. Therefore, socioeconomic indicators are associated with risk factors for dental caries [2,3]. Socially disadvantaged individuals also experience disadvantages with regard to health in general. The greater frequencies of disease in small population groups are known as polarization [4,5]. The association between the relative position each social group occupies and differences in the risk for various health conditions and in access to healthcare services makes social stratification a determinant of these conditions.

Social epidemiology has made great advances over the past three decades at a time when health inequalities have widened across countries. This situation challenges researchers to understand the social disparities in health to improve population health [6], despite the need to generate improved theoretical frameworks and the necessary data to test and refine them [7]. Recently, it was reinforced that social class or socioeconomic position, are not only a striking predictor of disease occurrence, but the associations reflects causal connections too [8].

The causal approach in dental caries was previously presented. Low socioeconomic status, low monthly household income and low educational level are associated with less access to dental services and oral hygiene products, poorer knowledge regarding oral health and oral hygiene and, consequently, a greater frequency and severity of dental caries [9].

Although a number of epidemiological studies have evaluated the associations between dental caries and socioeconomic indicators, no systematic reviews in the literature offer scientific evidence of such associations. The aim of the present study was to perform a systematic review to evaluate the associations between socioeconomic indicators and dental caries in adults by narrative synthesis. The hypothesis was that adults with worse socioeconomic indicators are more affected by dental caries. 

## 2. Methods

All epidemiological studies (cross-sectional, case-control, cohort and clinical trials) involving adult populations aged 19 to 60 years that reported etiological factors and/or the prevalence of dental caries or risk factors for dental caries were considered eligible for the present review. Study selection was conducted in two phases: (1) abstracts and titles were selected and (2) full texts of the selected titles were obtained and read to determine the final sample set. 

The epidemiological question investigated in this study was as follows: are adults with worse socioeconomic indicators more affected by dental caries than adults with better socioeconomic indicators? The socioeconomic indicators included any reference to schooling, income, type of occupation or employment, socioeconomic status, any population index, access/non-access to private dental practice and satisfaction with one’s income.

### 2.1. Search Strategy

Seven databases were systematically searched: MEDLINE using PubMed (www.pubmed.gov), The Cochrane Library (http://www.cochrane.org/index.htm), including Cochrane database for Systematic Reviews, Database of Abstracts of Reviews of Effectiveness, Cochrane Controlled Trials Register and Cochrane Review Methodology Database; Web of Science (http://www.isiknowledge.com), Controlled-Trial Database (http://controlled-trial.com), Clinical Trials-US National Institutes of Health (http://www.clinicaltrials.gov), the National Institute for Health and Clinical Excellence (http://www.nice.org.uk) and the Virtual Health Library (Bireme-Latin America; www.bireme.br). No restrictions were placed on the language or year of publication. Searches were performed in July 2010, and a new search was conducted in August 2012 in Medline to update the findings.

The following search strategy was used in the Medline: caries OR Dental Caries (Mesh) OR dental decay OR DMF index (Mesh) OR decayed teeth OR DMFS OR DMFT AND socioeconomic factors (Mesh) OR social class (Mesh) OR educational status (Mesh) OR educational level OR socioeconomic condition OR socioeconomic level OR socioeconomic determinant* OR social determinant* OR income (Mesh) OR poverty (Mesh) OR risk factors (Mesh) OR occupational class. In Medline, the search was limited to include only studies with subjects ≥19 years of age.

The following search strategy was used for Cochrane Library and Web of Science: caries OR Dental Caries (Mesh) OR dental decay OR DMF index (Mesh) OR decayed teeth) AND (socioeconomic factors (Mesh) OR social class (Mesh) OR educational status (Mesh) OR educational level OR socioeconomic condition OR socioeconomic level OR socioeconomic determinant* OR social determinant* OR income (Mesh) OR poverty (Mesh) OR risk factors (Mesh). 

The Virtual Health Library (Bireme) included both the Latin-American and Caribbean System on Health Science Information (Lilacs) and Brazilian Library of Dentistry (BBO) databases. In the Lilacs, BBO and Clinical Trials databases, two keywords were used at a time because these databases did not support the entire search strategy. In the Lilacs and BBO databases, keywords in Portuguese were also used. In the Controlled-Trial and National Institute for Health and Clinical Excellence databases, one keyword was used at a time. 

The search was conducted by three researchers (Simone Melo Costa, Maria de Lourdes Carvalho Bonfim and Carolina de Castro Martins). The studies were entered into the Reference Manager® programs, and a list was generated for analysis and selection. 

### 2.2. Selection of Studies and Data Extraction

Studies retrieved from the databases were selected after reading the abstracts and titles, following a calibration exercise with 10% of the studies read by three independent reviewers to determine inter-examiner agreement (Kappa: 0.68 to 0.97). Disagreements were resolved by consensus. The following were the inclusion criteria for the initial selection process: reviews, epidemiological studies with subjects between 19 and 60 years of age, studies addressing risk factors for dental caries and studies reporting socioeconomic indicators. No clinical trials were found by the searches although all caution was taken to try to find them. For this reason, no clinical trials are considered this in this review. Reviews were included, and their reference lists were searched in turn for any studies not retrieved by the electronic search. However, this process yielded no further studies. Initially, all studies addressing risk factors for dental caries were selected, even if socioeconomic indicators were not the main subject. This was a try to find hidden studies that reported socioeconomic indicators as confounders but in which they was not mentioned in the title nor in the abstract. In this fashion, more studies were selected for full text analysis than were expected to meet the review conditions and it was an effort to find important related studies.

**Figure 1:**
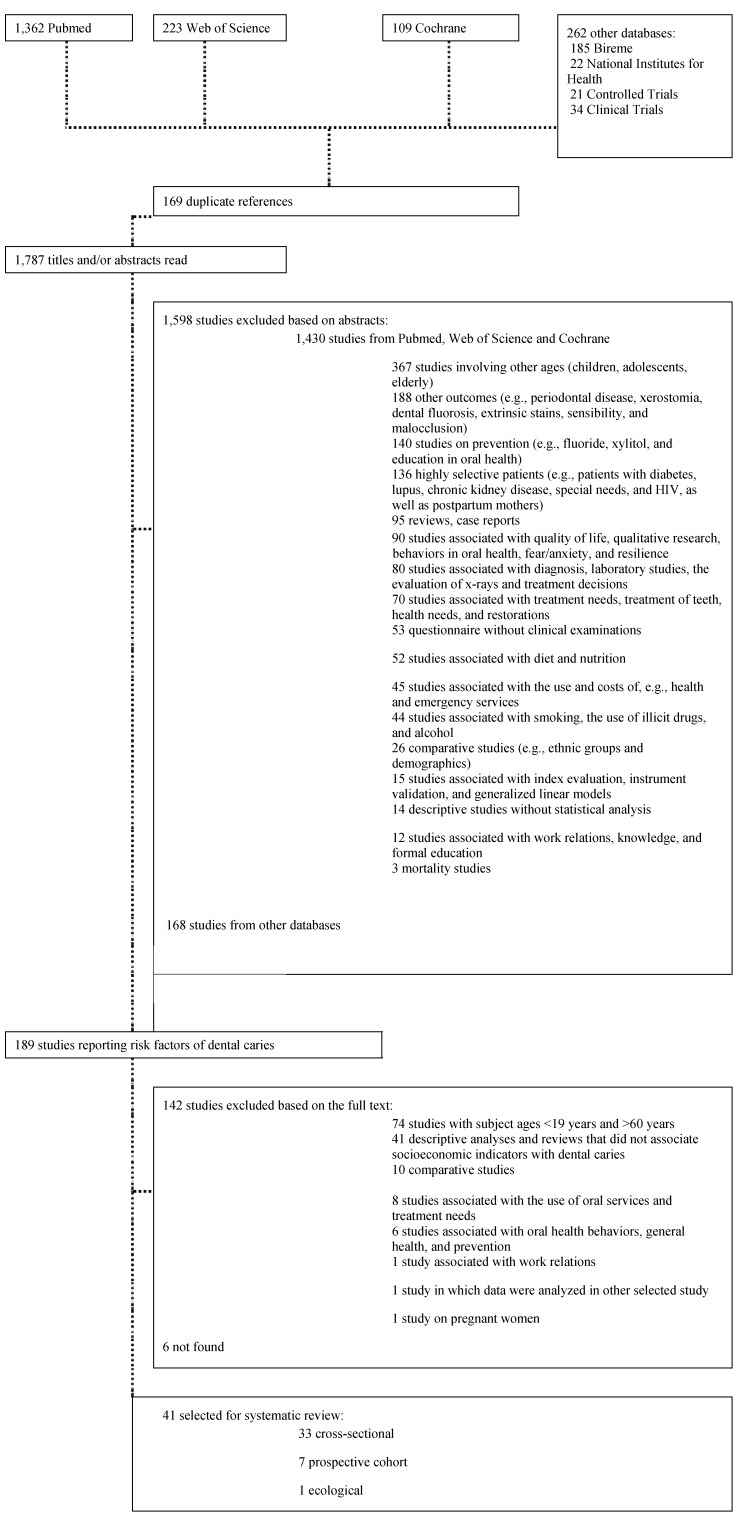
Flowchart of study selection.

The exclusion criteria are presented in Figure 1. Studies involving patients younger than 19 or older than 60 years were excluded; studies involving other outcomes (dental fluorosis, periodontal disease and others); studies evaluating fluorides, xylitol or educational programs; case reports; studies reporting oral health related quality of life as main outcome; laboratorial and diagnostic studies (X ray or clinical examination); studies evaluating treatment needs, health services and satisfaction with dental services; studies related to diet and nutrition; studies that did not perform clinical examination to evaluate dental caries; studies reporting risk factors related to alcohol, tobacco and drugs use; studies of validation methods, development of new methods or indexes; studies that selected groups based on demographic location (rural and urban or ethnic groups); studies without statistical analysis (only frequency data); mortality groups or high specific groups as diabetics, pregnancy and others; and reviews not related to dental caries.

The full texts were obtained from the selected studies. Seventeen authors were contacted by email, and eleven authors answered these emails [10,11,12,13,14,15,16,17,18,19,20]. Among the 189 studies, the full texts for only six could not be found [21,22,23,24,25,26]. The full texts of the studies were read by two independent reviewers (Simone de Melo Costa and Maria de Lourdes Carvalho Bonfim) following a calibration exercise with 10 studies. Disagreements were resolved by consensus. In this phase, reviews and studies that did not report statistical tests for dental caries and socioeconomic indicators (*i.e.*, studies that reported only prevalence data or descriptive data) and studies with populations involving individuals younger than 19 years or older than 60 years were excluded. Data extraction was conducted by two independent reviewers (Simone de Melo Costa and Maria de Lourdes Carvalho Bonfim). It was developed a form for data extraction and all studies were evaluated considering this form. 

### 2.3. Quality Assessment

Two independent reviewers (Simone de Melo Costa and Maria de Lourdes Carvalho Bonfim) evaluated the quality of the studies using the Newcastle-Ottawa scale for cohort studies. Cross-sectional studies were evaluated using the Newcastle-Ottawa scale modified for case-control studies [27]. Study quality was rated on a scale from 1 (very poor) to 9 (high). Disagreements were resolved by consensus. The included ecological study was evaluated as a cross-sectional study.

### 2.4. Data Synthesis

The extraction of the data was based on the study design, population characteristics, the measures used for dental caries and the type of socioeconomic indicators used. A high degree of heterogeneity was found among the methodologies and types of socioeconomic indicators used. Dental caries and socioeconomic variables had several categories and cut points. Therefore, it was not possible to group data for meta-analysis. Instead, narrative synthesis was conducted in this review.

## 3. Results

A total of 1,957 potentially relevant records were found in the seven databases, 169 of which were duplicated. Thus, the abstracts of 1,787 studies were read. A total of 1,598 references were excluded based on the abstracts, and 189 were selected for full-text analysis, 41 of which were selected for inclusion. The systematic review comprised 33 cross-sectional studies, seven prospective cohort studies and one ecological study (Figure 1). No clinical trials were found that evaluated the association between dental caries and socioeconomic indicators.

### 3.1. Quality Assessment

Agreement between the reviewers on each item of the Newcastle-Ottawa scale was 100%. The characteristics of the studies are displayed in Table 1, Table 2, Table 3. Regarding quality, cross-sectional studies received between five and eight points [12,16,17,18,28,29,30,31,32,33,34,35,36,37,38,39,40,41,42,43,44,45,46,47,48,49,50,51,52,53,54,55,56] (Table 1), the ecological study received five points [57], one prospective cohort study received the highest score of nine points [58] and three prospective cohort studies received eight points each [59,60,61]. The studies by Bille [62] and Holst and Schuller [63] received six points and Hahn *et al.* [64] received seven points. Although these were originally cohort studies, the results of the socioeconomic data were derived from a cross-sectional presentation (Table 2).

**Table 1:** Study characteristics and results reported from cross-sectional studies.

Authors, year	Country	Location of data collection	Total number of respondents (Final sample size)	Subjects’ age (years)	Caries index	Socioeconomic indicator	Other measures	Association between socioeconomic indicators and dental caries	Newcastle-Ottawa scale
Nikias *et al.*, 1975 [28]	USA	Clinical setting	1,290 (1,122)	19+ years	Decayed teeth	Status: poverty and non-poverty	Gender, age, edentulousness, soft tissue lesions, gingival status, oral hygiene levels, visit to dentist, frequency of brushing	Poverty and more dental caries	5 (9)
					Mean			(*p* < 0.05) *	
					Number of decayed teeth: none, 1-2, 3 or more				
Hansen, 1977 [29]	Norway	Clinical setting	177 (117)	35 years	DMFT	Years of schooling	Gender	Less schooling and more dental caries	6 (9)
					Decayed teeth	(≤10 and >10)			
					Mean			(*p* > 0.05) *	
Meyer *et al.*, 1983 [30]	Portugal	Clinical setting	73 (73)	21 to 30 years	DMFT	Lower socioeconomic status (manual laborers) and higher socioeconomic status (the first three classes of students graduating from the new dental school in Lisbon)	Periodontal Index, gender, oral hygiene	Lower socioeconomic status and lower DMFT =15.9 ± 6.3, 10.0 ± 5.0	6 (9)
					DMFS				
					Mean				
								DMFS = 42.6 ± 25.0, 24.1 ± 15.3	
								(*p* < 0.05) *	
Tervonen *et al.*, 1991 [31]	Finland	Not reported	1,600 (883)	25, 35, 50 years	Number of decayed teeth	Years of education: university level, college, vocational school, compulsory, secondary school, less than 6 years of junior high school.	Periodontal disease, age, gender, number of teeth, regularity of attendance for treatment, attitude to preservation of teeth, and others.	Less schooling and more decayed teeth	7 (9)
								(*p* < 0.05) **	
					(D < 7 or ≥7)				
								OR = 1.12 (1.03–1.20)	
Marcenes & Sheiham, 1992 [32]	Brazil	Subjects’ homes	164 (164)	35 to 44 years	DMFS	Socioeconomic status by ABA-ABIPEME	Periodontal status, age, frequency of brushing, sugar consumption, frequency of dental care, mental demand of work, marital quality, and others	Lower socioeconomic status and greater DMFS	7 (9)
					Mean				
								(*p* < 0.05) *	
								(*p* > 0.05) **	
Eriksen *et al.*, 1996 [33]	Portugal	Clinical setting	322 (196)	30 to 39 years	Number of decayed surfaces-DS	Social class: class 1, class 2 and class 3	Smoking, psychological status, eating between meals, tooth cleaning (OHI-S), brushing frequency, interdental cleaning, use of fluoridated toothpaste, gender, regular dental visits, and others	Lower social class and more decayed surfaces	6 (9)
						Years of schooling: ≤10 and >10			
								(*p* > 0.05) *	
								DS = 12.6 ± 11.1, 12.4 ± 11.6, 15.7 ± 13.2	
					Mean				
								Less schooling and more decayed surfaces	
								(*p* > 0.05) *	
								DS = 14.5 ± 12.7, 11.7 ± 10.8	
Berset *et al.*, 1996 [34]	Norway	Dental Faculty, University of Oslo	178 (121)	35 years	DMFS	Social class: low, medium, high	Oral hygiene, brushing frequency, use of fluoride toothpaste, saliva secretion, mutans strep., gender, dental visits, and others	Low social class and carious surfaces	7 (9)
					Mean				
						Years of schooling: ≤12 and >12			
								(*p* < 0.001)*	
								(3.4 ± 4.1, 1.1 ± 1.1, 1.3 ± 1.5)	
						Finances: no/minor/major problems			
								<12 years of schooling and higher number of carious surfaces	
								(*p* < 0.01) *	
								(3.1 ± 3.8, 1.4 ± 1.8)	
								Unsatisfied with own economic status and carious surfaces	
								(*p* < 0.01) *	
								(1.5 ± 2.0, 1.6 ± 1.3, 4.4 ± 4.5)	
								Variation in decayed surfaces could be explained by social class, economic condition and others	
								(R^2^ = 0.35) **	
Hescot *et al.*, 1997 [35]	France	Exam carried out on clusters (transport-able dental chair)	1,000 (1,000)	35 to 44 years	DMFT and decayed teeth (DT)	Occupational group: high, medium, low (derived from the combination of occupational activity, educational level and household income)	Gender, residence (urban, rural), one or more surface fillings, treatment need (pulp care, extraction or other treatment)	Lower occupational group and more decayed teeth	6 (9)
					Mean				
								(*p* > 0.05) *	
								DT = 0.9 ± 1.8, 1.2 ± 2.2, 1.3 ± 2.0	
Schuller, 1999 [36]	Norway	Not reported	Evaluation of two sample groups in 1983 (945 (796)) and 1994 (702 (454))	23 to 24 years	Decayed and filled surfaces (DFS)	Years of education: ≤12 and ≥13	Gender, residence (urban, rural), time since last dental visit, type of dental clinic, use of dental service, treatment received, oral hygiene score, and others	Less schooling and more decayed and filled surfaces in both years analyzed	6 (9)
					Mean				
								(*p* < 0.05) *	
								(1983 = 40.7, 37.0)	
								(1994 = 22.3, 15.6)	
Sgan-Cohen *et al.*, 1999 [37]	Israel	Clinical setting of military institute	1,300 (1,084)	25 to 44 years	DMFT	Years of education: <12, 12 and >12	Gender, age	Less schooling and more decayed teeth	6 (9)
					Decayed teeth (DT)				
					Mean				
								(*p* < 0.001) *	
								DT = 1.75 ± 2.4, 1.53 ± 2.2, 0,89 ± 1.4	
								(*p* < 0.001) **	
								Number of years of education with the D component-Rb = −0.16	
Unell *et al.*, 1999 [38]	Örebro and Östergötland(Sweden)	Not reported	6,343 (513)	50 years and older	Decayed and filled teeth	Occupational status: white-collar workers in leading positions, white-collar workers, entrepreneurs, blue-collar workers	Marital status, gender, residence (rural, town, city), working hours, general self-perceived health, mouth dryness, tobacco user, satisfied with dental care, use of dental services, and others	Lower occupational status and more decayed teeth	7 (9)
					Decayed teeth (DT)				
						Education: college, high school/grammar school,			
								(*p* < 0.05) **	
						secondary education, primary education			
								Less schooling and more decayed teeth	
								(*p* < 0.05) **	
Brodeur *et al.*, 2000 [40]	Canada	Not reported	4,742 (2,110)	35 to 44 years	DMFT/DMFS	Family income:	Last visit to a dentist, gender, number of teeth in mouth, language spoken, age, area of residence (metropolitan, urban, rural), and others	Lower income and more decayed	7 (9)
					Decayed surfaces (crown and root) DS	less than $30,000, $30,000 to $59,999, $60,000 and greater (ref.)			
					Mean			(*p* < 0.05) *	
					Number of decayed surfaces: ≤3 and ≥4	Education: primary/high school, vocational training/college, university (ref.)		<$30,000-DS = 2.6 ≥$60,000-DS = 0.9	
								(*p* < 0.05) **	
								OR = 3.8 (2.19–6.48)	
								2.9 (1.72–4.86)	
								Less schooling and more decayed surfaces (*p* < 0.05) *	
								Primary-DS = 2.3	
								University-DS = 1.3	
								(*p* > 0.05) **	
								OR = 1.2 (0.79–1.81)	
								1.1 (0.69–1.71)	
Doughan *et al.*, 2000 [41]	Lebanon	Clinical setting	401 (401)	35 to 44 years	DMFT	Socioeconomic status: low, middle, high	Gender, residence (urban, rural), treatment need	Worse socioeconomic status and more decayed teeth	7 (9)
					Decayed teeth- DT				
					Mean				
								(*p* < 0.05) *	
								DT = 5.7 ± 5.7, 4.0 ± 4.7, 2.2 ± 2.8)	
Skudutyte *et al.*, 2000 [42]	Lithuania	Clinical setting	767 (380)	35 to 44 years	DMFT/DMFS	Education: low, medium, high	Gender, residence (urban, rural), fluoridated water, oral hygiene index (OHI-S)	Less schooling and more decayed teeth	6 (9)
					Decayed teeth-DT				
					Decayed surfaces				
					Mean/Median				
								(*p * < 0.05) *	
								DT = 3.0, 2.0, 1.0	
Paulander *et al.*, 2003 [45]	Sweden	Clinical setting	588 (588)	35 and 50 years	DMFS	Education: low and high	Number of teeth, probing attachment level, periodontal treatment needs, prevalence of dental caries, dietary habits, and others	Less schooling and more DS	6 (9)
					Decayed surfaces-DS				
					Mean				
								(*p* > 0.05) *	
								35 years-	
								DS = 1.3 (−0.2–2.7)	
								0.5 (−0.3–1.1)	
								50 years-	
								DS = 0.4 (0.2–0.6)	
								0.4 (0.1–0.7)	
Senna *et al.*, 2005 [47]	Italy	Clinical setting of a military institute	3,661 (2,908)	19 to 25 years	DMFT	Educational level: completed college or graduate school, high school, secondary school and primary school	Gingival and periodontal status	Less schooling and more decayed teeth	5 (9)
					Decayed teeth-DT				
					Mean				
								(*p* < 0.05) *	
								DT = 0.7 ± 1.2, 1.0 ± 1.4, 1.5 ± 1.9, 1.9 ± 1.9	
Badel *et al.*, 2006 [12]	Croatia	Clinical setting of a military institute	248 (248)	19 to 29 years	DMFT	Schooling in 3 categories: primary, secondary, university	Sugar intake	Less schooling and more decayed teeth	6 (9)
					Decayed teeth				
					(quartile cutoffs: Q25, Q50 and Q75%)				
								(*p* < 0.05) *	
								Q75% = 5, 4, 1.5	
Julihn *et al.*, 2006 [48]	Sweden	Clinical setting	800 (696)	19 years	DMFS	Educational level of father: ≤9 years, 10–12 years, >12 years	Gender, chronic disease, country of birth, years living in Sweden, oral hygiene, attitude toward dental care, dental fear, gingival bleeding index (GBI), and others	Less education of the father and more decayed surfaces	7 (9)
					<10 and ≥10				
						Occupational status of mother and father: unemployed, laborer, white-collar worker			
								(*p* < 0.01) *	
								(*p* > 0.05) **	
								Worse occupation of the father and more decayed surfaces	
								(*p* < 0.01) *	
								(*p* > 0.05) **	
								Worse occupation of the mother and more decayed surfaces	
								(*p* < 0.01) *	
								(*p* > 0.05) **	
Varenne *et al.*, 2006 [49]	Burkina Faso	Subjects’ homes	493 (493)	35 to 44 years	DMFT	Occupation: shop keeper, government employee, smallholder/craftsman, housewife, farmer/breeder(ref.)	Gender, ethnical group, dental visits, use of chewstick, consumption of fresh fruits, location (urban, rural), and others	Government employee and higher DMFT	7 (9)
					Mean				
					Absence/presence of caries				
						Educational level: high, moderate, low			
								(*p* < 0.05) **	
								OR = 5.26	
								High educational level and higher CPOD	
								(*p* < 0.05) **	
								OR = 2.99	
Hessari *et al.*, 2007 [51]	Iran	Non-specific	8,301 (8,301)	35 to 44 years	DMFT	Level of education: illiterate, low, medium, high	Gender, place of residence (urban, rural)	Less schooling and more decayed teeth	7 (9)
					Decayed teeth-DT				
					Mean				
								(*p* < 0.05) *	
								Men-DT = 2.7 ± 2.7, 2.8 ± 2.6, 2.4 ± 2.7, 1.9 ± 2.4	
								Women-DT = 2.8 ± 2.7, 2.8 ± 2.8, 1.9 ± 2.4, 1.9 ± 2.6	
Roberts-Thomson & Stewart, 2008 [52]	Australia	Clinical setting	1,261(644)	20 to 24 years	DMFS	Tertiary education (yes or no)	Gender, country of birth, living at home, visit in last 2 years, usual reason for visit, site of last visit, brushing, current smoker, alcohol use, and others	Less schooling and more cavitated caries (*p* > 0.05) *	7 (9)
					Decayed surfaces-DS	Employed (yes or no)			
					Mean	Income (<$20,000 or $20,000 or more)			
					DMFS modified by Protocol of US National Institute of Dental Research: precavitated decayed surface				
						Government benefits (yes or no)			
								DS = 0.64 ± 3.00, 1.06 ± 2.55	
								Unemployed and more cavitated caries (*p* < 0.05) *	
					Mean				
								DS = 0.64 ± 1.47, 1.16 ± 5.83	
								(*p* < 0.05) **	
								Lower income and more cavitated caries (*p* > 0.05) *	
								DS = 0.94 ± 3.18, 0.85 ± 2.18	
								Receiving benefits and more cavitated caries (*p* < 0.05) *	
								DS = 1.68 ± 4.94, 0.70 ± 1.76	
								(*p* > 0.05) **	
Skudutyte-Rysstad *et al.*, 2009 [54]	Norway	Clinical setting	149 (149)	35 years	DMFT	Education: no university or university	Gender, marital status, region of birth, dental anxiety score, frequency of brushing, use of dental floss, dental visits, time since last dental visit, smoking	Less schooling and more decayed surfaces	6 (9)
					DMFS	Household income (NOK/year):			
					Decayed surfaces on dentin (≥2 and <2)-DS	≤299,000 (low),			
						300,000–599,000 (medium) and ≥600,000 (high)			
								(*p* < 0.05) *	
								Lower income and more decayed surfaces	
								(*p* < 0.05) *	
								(*p* < 0.05) **	
								DS-OR = 4.5 (1.9–10.2)	
Brennan *et al.*, 2010 [17]	Australia	Clinical setting	879 (709)	45 to 54 years	DMFT	Household income: under AU$30,000, AU$30,000–$60,000, over AU$60,000	Gender, place of birth, dental knowledge of tooth decay prevention	Lower income and more decayed teeth	7 (9)
					Decayed teeth-DT				
					Mean				
								(*p* < 0.01) *	
								DT = 0.8 ± 0.13, 0.4 ± 0.07, 0.2 ± 0.03	
								(*p* < 0.01) **	
Geyer *et al.*, 2010 [16]	Germany	Not reported	1,779 (925)	35 to 44 years	DMFT	Income in categories: highest, second highest, intermediate, second lowest and lowest;	Gender, age (years)	Lower income and higher DMFT	7 (9)
					(≤21 and >21)				
						Educational level: 12–13 years, 10 years and 8–9 years of schooling		(*p* < 0.05) *	
								OR = 3.74 (1.66–8.46)	
						Cumulative effects: income + educational level l = highest socioeconomic positions, intermediate positions, lowest positions			
								(*p* < 0.05) **	
								OR= 2.34 (1.00–5.55)	
								Less schooling and higher DMFT	
								(*p* < 0.05) *	
								OR = 3.75 (1.99–7.05)	
								(*p* < 0.05) **	
								OR = 2.95 (1.52–5.74)	
								Socioeconomic status - lowest positions: income + education level and higher DMFT	
								(*p* < 0.05)**	
								OR = 6.06 (2.06–17.87)	
Celeste *et al.*, 2011 [55]	Brazil	Subjects’ homes	22,839 (20,695)	35 to 44 years	DMFT	Gini (quartile), municipal income (quartile), household income (based on minimum wage)	Gender, age, place of residence (urban, rural), last dental visit., edentulism, prevalence of periodontal attachment loss >8 mm	More iniquity results in more decayed teeth	7 (9)
					Untreated dental caries				
					Mean				
								(*p* < 0.05) **	
Brennan *et al.*, 2011 [18]	Australia	Clinical setting	879 (709)	45 to 54 years	DMFT	Household income (under $80,000+ and <$80,000	Dental visit pattern, dental self-care (tooth brushing)	Lower income and more decayed teeth	7 (9)
					Decayed teeth-DT				
					Mean	Education: tertiary and secondary			
								(*p* < 0.01) *	
								DT = 0.1 ± 0.03, 0.5 ± 0.05	
								(*p* < 0.001) **	
								Income $80,000+	
								Beta = −0.27	
								Lesser schooling and more decayed teeth	
								(*p* < 0.01) *	
								DT = 0.2 ± 0.03, 0.5 ± 0.06	
								(*p* < 0.01) **	
								Education tertiary Beta = −0.25	
Chandra *et al.*, 2011 [56]	India	Not reported	1,198 (1,187)	19 to 57 years	DMFT	Socioeconomic status(SES): upper, upper middle, lower middle, upper lower, lower	Gender, periodontal status, oral pre-malignant, malignant lesions, demographic profile, *etc.*	Lower socioeconomic status and more decayed teeth	6 (9)
					Decayed teeth-DT				
					Mean				
						(Modified Kuppuswamy scale were based on the 1988–1989)			
								(*p* < 0.001)*	
								Upper-DT = 0.07 (±0.32)	
								Lower DT = 0.96 (±2.06)	

* Bivariate analysis; ** multivariate analysis.

**Table 2:** Study characteristics and results from seven prospective cohort studies.

Authors, year	Country	Location of data collection	Total number of respondents (Final sample size)	Subjects’ age (years)	Caries index	Socioeconomic indicator	Other measures	Association between socioeconomic indicators and dental cariess	Newcastle-Ottawa scale
Bille, 1980 [62]	Denmark	Subjects’ homes	389 (313)	Data evaluated at 20 years of age (cross-sectional)	DMFS	Subjects’ socioeconomic status and parents’ socioeconomic status by occupation:	Gender, dental visits	Lower socioeconomic status of parents and higher DMFS	6 (9)
					Mean				
								(*p* > 0.05)*	
						low (unemployment, unskilled and semiskilled occupations);		Lower socioeconomic status of subject and higher DMFT	
								(*p* < 0.01)*	
						medium (non-manual and manual skilled occupations);			
						and high (intermediate non-manual, administrative and professional occupations)			
Bjertness *et al.*, 1992 [60]	Norway	Not reported	116 (81) Data evaluated in 1973 and 1988	35 and 50 years	Decayed	Years at school:	Alcohol, exercise, smoking, psychological status, sugar between meals, teeth cleaning, use of fluoride, interdental cleaning, allergies, medications, regular dental visits, and others	Less schooling and more decayed teeth	8 (9)
					teeth-DT	≤10 and >10			
					Mean	Social class: class 1, class 2, class 3;			
								(*p* > 0.05) *	
						Satisfaction with own finances: unsatisfied, satisfied;		DT = 1.27 ± 0.452, 1.22 ± 0.417	
								Lower social class and more decayed teeth	
								(*p* > 0.05) *	
								DT = 1.46 ± 0.522, 1.19 ± 0.398, 1.22 ± 0.428	
								Dissatisfaction with finances and more decayed teeth	
								(*p* > 0.05) *	
								DT = 1.14 ± 0.378, 1.24 ± 0.432	
Hahn *et al.*, 1999 [64]	Germany	Clinical setting	300 (298)	50 to 60 years	DMFT	Education: low, middle, high	Gender, low-sugar nutrition, use of fluoride, dental attendance, reason for last visit, smoking habits, and others	Less schooling and decayed roots	7 (9)
			in the beginning of the study		Decayed root				
								(*p* > 0.05) **	
			(cross-sectional)					Education-	
								Β = 0.0129	
Gilbert *et al.*, 2001 [59]	USA	Not reported	873 (723) (24 months)	45 years and older	Decayed or filled root surface	High school graduate (yes, no)	Regular dental visits; flosses daily or more often; flosses, but less than daily; area of residence (rural, urban)	Less schooling and more new caries or restorations	8 (9)
					(new root decay only; new root filling(s) only; both new decay and new filling(s) or neither)	Income ( able to pay, but with difficulty or not able to pay)			
								(*p* < 0.05) *	
								Not able to pay and more new caries	
								(*p* < 0.05) **	
								OR = 2.5	
Thomson *et al.*, 2004 [58]	New Zealand	Not reported	922 (838)	Dental exam for caries at ages 5 and 26 years	DMFS	Socioeconomic trajectory (evaluated at 5 and 26 years of age): high-high, low-high, high-low, low-low	Tooth loss, periodontal disease, self-care, brush less than once daily, gender, time spent living in fluoridated area	Low-low and high-low socioeconomic trajectories and more decayed surfaces	9 (9)
					Decayed surfaces-DS				
					Decayed/filled surfaces				
					Loss due to caries				
					Mean				
								(*p* < 0.05) **	
								Mean DS	
								High-high = 1.26	
								Low-high = 1.61	
								High-low = 1.94	
								Low-low = 2.05	
Holst & Schuller, 2011 [63]	Nord-Trondelag	Clinical setting	Two Birth-cohorts in age groups between 35–44 from 1983 to 2006	Age-group 35–44 years	DMFT	Education: quartile (shortest education, second shortest education, second longest education, longest education)	Age	1983 -Less schooling and more DS	6 (9)
					DMFS				
								(*p* < 0.05) *	
								2006- education and DMFS (*p* > 0.05) *	
			Year 1983 = 500 (300)		Decayed surfaces-DS				
			1994 = 350 (135)		Decayed teeth-DT				
					Mean				
			2006 = 250 (158)						
			(cross-sectional results over 33 years)						
Shearer *et al.*, 2012 [61]	New Zealand	Not reported	Birth cohort of 1,037 children born at the queen Mary Hospital	32 years	DMFS	Socioeconomic (SES): low, medium, high	Sex, use of dental services, smoking status, familial risk, plaque trajectory	Less SES at age 32 and more DMFS	8 (9)
					Decayed surface-DS				
								(*p* > 0.05) **	
								Low SES-RR = 1.15 (0.95–1.40)	
			932 dentally examined at age 32 years (626—had both parents interviewed (complete information)		Mean				
					DMFS >20			Medium SES	
					DMFS = 12			RR = 1.05 (0.88–1.26)	

* Bivariate analysis; ** multivariate analysis.

**Table 3:** Study characteristics and results from ecological study.

Authors, year	Country	Location of data collection	Total number of respondents (Final sample size)	Subjects’ age (years)	Caries index	Socioeconomic indicator	Other measures	Association between socioeconomic indicators and dental caries	Newcastle-Ottawa scale	
Bernabe *et al.,* 2009 [57]	18 countries	National statistics on dental caries experience obtained from WHO Oral Health Country/Area Profile Programme	Ecological data from the 50 richest countries in the world (18 included in the analysis)	35 to 44 years	DMFT	Gross domestic product *per capita*;	Caries index, restorative index, treatment index	Worse Gini coefficient and more decayed teeth	5 (9)	
					Decayed teeth-DT					
						Gross national income *per capita* in 2000 (in dollars);				
								(*p* > 0.05) *		
		(Surveys conducted between 1995 and 2005)								
						Gini coefficient				

* Bivariate analysis.

### 3.2. Study Location and Language

The language of the cross-sectional studies was predominantly English, although the studies were conducted in different countries, namely Germany, Finland, Italy, Burkina Faso (Africa), Norway, Brazil, the United States, Australia, Sweden, Israel, Lithuania, Portugal, Denmark, Iran, China, Lebanon and Canada. One cross-sectional study was published in Croatian and was translated prior to analysis [12] (Table 1 and Table 2). The only ecological study included in the present study involved the analysis of health data from 18 different countries [57]. All studies were published between 1975 and 2012.

### 3.3. Population Characteristics

The studies involved populations in age groups between 19 and 60 years. In three studies, the participant ages were defined by a mean of 21 years [43], a minimum of 19 years [28], and a minimum of 50 years [38]. 

Twenty-five studies were population-based studies with randomization involving men and women, 17 of which defined the sample group prior to conducting the study [16,17,18,31,34,35,36,39,40,41,44,45,48,49,51,53,55]. Only nine studies reported that the sample group was representative of the population studied [16,34,39,40,41,48,51,53,55]. 

The studies by Brennan *et al.* [17,18] reported the analysis of data from a single epidemiological survey conducted in the city of Adelaide (southern Australia) in 2004–2005. However, the variables differed with regard to categorization. The studies by Celeste *et al.* [53,55] also included the analysis of data from a single epidemiological survey conducted in 2002–2003, with the participation of 330 municipalities of Brazil.

Five studies evaluated samples of men recruited at a military base (convenience samples) [12,37,43,46,47]. The study by Sgan-Cohen *et al.* [43] included data from exams performed in consecutive years (1994, 1995, 1996 and 1997), with a total of 7,139 military participants.

The cross-sectional study by Schuller [36] analyzed data on the population of Oslo (Norway) from 1983 and 1994, describing statistically significant associations between the socioeconomic indicators and dental caries in both years. The ecological study addressed dental caries in 18 of the 50 richest countries in the world, namely Australia, Austria, Canada, Denmark, Finland, France, Germany, China, Ireland, Israel, Italy, Japan, Singapore, Slovenia, Spain, Sweden, the United Kingdom and the United States [57] (Table 3). The number of participants submitted to oral exams in the 38 studies ranged from 73 [30] to 20,695 [55].

### 3.4. Measures of Dental Caries and Data Collection

Different indices were identified, such as decayed, missing and filled teeth (DMFT); decayed, missing and filled surfaces (DMFS); decayed and filled teeth (DFT) and decayed and filled surfaces (DFS). Different diagnostic criteria for dental caries, such as criteria established by the World Health Organization (WHO) and criteria proposed by the US National Institute of Dental Research, were also identified. The following were the parameters used for the assessment of dental caries:

√ Mean or median of the DMFT index and/or separate components [16,17,18,28,29,30,33,35,37,39,40,41,42,43,44,46,47,49,50,51,53,54,55,56,57,60,63];
√ Mean or median of the DMFS index and/or separate components [30,32,34,36,39,40,45,52,54,61,62,63];
√ Mean number of surfaces with non-cavitated caries [52];
√ Mean number of teeth with root decay [64];
√ Mean number and percentage of functional teeth [28];
√ Quartiles of total number of decayed teeth and DMFT index (25, 50 and 75%) [13];
√ Total number of decayed teeth and total number of decayed/filled teeth [38];
√ Number of decayed teeth, categorized as none, one to two decayed teeth and three or more decayed teeth [28];
√ DMFT, categorized as ≤21 and >21 [16];
√ DMFS, categorized as <10 and ≥10 [48];
√ DMFS, categorized as >20 [61];
√ DMFS, categorized as 12 [61];
√ Number of decayed surfaces, categorized as <4 and ≥4 [40];
√ Number of decayed teeth, categorized as ≥7 and <7 [31];
√ Number of decayed surfaces, categorized as ≥2 and <2 [54];
√ Absence of new carious lesions or new restorations [59];
Number of decayed and filled root surfaces [40,59].

Nineteen studies reported using the WHO criteria for the oral exam [16,30,32,34,35,37,39,40,41,42,43,44,46,47,49,51,53,54,55]. Regarding the exam location, the clinical setting was described in 18 studies [12,17,18,28,29,30,33,37,39,42,45,47,48,50,52,54,60,63,64]. In all studies, the exams for the diagnosis of dental caries were performed by dentists, with the exception of one study, in which the exams were performed by a dental hygienist [28].

In eight studies, X-ray exams were performed in tandem with clinical exams for the diagnosis of dental caries [29,33,34,45,48,54,60,62]. In the study by Bille [62], radiographs were taken at the homes of the subjects using a portable device. Only two studies reported using X-ray exams to calibrate the oral health professionals in their evaluation of bitewings [38,62]. However, it is unclear in the study by Unell *et al.* [38] whether the X-ray exam was used as a complementary exam for the diagnosis of carious lesions. 

Regarding the calibration of the researchers for the clinical exam of the teeth, only 19 studies (46.34%) described the Kappa index value or percentage of intra-examiner and/or inter-examiner agreement [32,34,35,39,40,41,42,43,44,47,48,51,52,53,54,55,60,62,63]. Kappa results ranged from 0.61 to 0.98. 

### 3.5. Socioeconomic Indicators and Other Variables

Different socioeconomic criteria were considered in the studies, demonstrating considerable diversity among the indices and criteria employed (Table 1, Table 2, Table 3): schooling, literacy rate, school frequency, educational level (in years of study), socioeconomic status, social status, inequity regarding municipal revenue, social class, household income, income *per capita*, government benefits, satisfaction with income, occupation, employed population, unemployment and community indices, such as the Gini coefficient, which measures the degree of inequality in the distribution of individuals based on income *per capita* (ranging from 0 (absence of inequality) to 1 (maximal inequality)). 

### 3.6. Statistical Analysis of Associations between Dental Caries and Socioeconomic Indicators

Twenty one out of the 41 studies employed multivariate statistical analyses, whereas 20 studies (48.78%) only employed bivariate analyses with no adjustments for confounding variables. For the 21 studies that employed multivariate analyses, only 11 studies presented the results of both bivariate and multivariate analyses (Table 1, Table 2, Table 3). Periodontal status, visits to the dentist, smoking habits, oral hygiene habits (brushing frequency, use of dental floss), gender, age and place of residence (urban or rural area) were used as confounding variables (Table 1, Table 2, Table 3). 

Fourteen studies used more than one socioeconomic variable, such as income and schooling, to assess associations with dental caries. Table 4 displays the quantitative distribution of the analyses performed on socioeconomic indicators by multivariate analysis and the associations: positive (+) (worse socioeconomic indicator associated with a greater severity of dental caries; 95% CI does not include 1.0 or *p* < 0.05); negative (–) (worse socioeconomic indicator associated with a lesser severity of dental caries; 95% CI does not include 1.0 or *p* < 0.05); and null (socioeconomic indicator not associated with severity of dental caries; 95% CI including 1.0 or *p* > 0.05). Besides 95% CI *p*-value was also considered because some studies reported results in *p*-value instead of 95% CI. The evaluation of the results of the analysis considered associations between socioeconomic determinants and the number of decayed or cavitated teeth. In the absence of the latter indicator, the evaluation considered the results of the DMFT, DMFS or other parameters used to determine the association with dental caries. 

**Table 4:** Quantitative distribution of statistical analyses and type of association (positive (+), negative (–) or null (#)) according to socioeconomic indicators.

Socioeconomic indicator	Multivariate analysis: Socioeconomic indicator and dental cariesOR (95%CI) or Beta or R^2^ or RR		
Type of association *	+ (95% CI does not include 1.0 or *p* < 0.05)	− (95% CI does not include 1.0 or *p* < 0.05)	# (95% CI includes 1.0 or *p* > 0.05)
**SCHOOLING**			
**Schooling Subject’s**			
Tervonen *et al.*, 1991 [31]	OR = 1.12 (1.03–1.20)		
Sgan-Cohen *et al.*, 1999 [37]	R = −0.16 (*p* < 0.001)		
Hahn *et al.*, 1999 [64]			(*p* > 0.05)
Unell *et al.*, 1999 [38]	(*p* < 0.05)		
Brodeur *et al.*, 2000 [40]			OR = 1.2 (0.79–1.81)
Sgan-Cohen *et al.*, 2000 [43]	(*p* < 0.001)		
Varenne *et al.*, 2006 [49]		OR = 2.99 (*p* < 0.05)	
Geyer *et al.*, 2010 [16]	OR = 2.95 (1.52–5.74)		
Brennan *et al.*, 2011 [18]	Β = −0.25 (*p* < 0.01)		
**Schooling Father’s**			
Julihn *et al.*, 2006 [48]			(*p* > 0.05)
**SUBJECT’S INCOME **			
Brodeur *et al.*, 2000 [40]	OR = 3.8 (2.19–6.48)		
Gilbert *et al.*, 2001 [59]	OR = 2.5 (*p* < 0.05)		
Brennan *et al.*, 2007 [50]	(*p* < 0.001)		
Skudutyte-Rysstad *et al.*, 2009 [54]	OR = 4.5 (1.9–10.2)		
Brennan *et al.*, 2010 [17]	(*p* < 0.01)		
Geyer *et al.*, 2010 [16]	OR = 2.34 (1.00–5.55)		
Brennan *et al.*, 2011 [18]	Beta = −0.27 (*p* < 0.001)		
**OCCUPATION/JOB**			
**Occupation/Subject’s job **			
Unell *et al.*, 1999 [38]	(*p* < 0.05)		
Varenne *et al.*, 2006 [49]		OR = 5.26 (*p* < 0.05)	
Roberts-Thomson *et al.*, 2008 [52]	(*p* < 0.05)		
**Occupation/Father’s job **			
Julihn *et al.*, 2006 [48]			(*p* > 0.05)
**Occupation/Mother’s job **			
Julihn *et al.*, 2006 [48]			(*p* > 0.05)
**SOCIOECONOMIC STATUS-SES**			
**SES Subject’s**			(*p* > 0.05)
Marcenes & Sheiham, 1992 [32]	R^2 ^= 0.35		
Berset *et al.*, 1996 [34]	OR = 6.06 (2.06–17.87)		
Geyer *et al.*, 2010 [16]			
Shearer *et al.*, 2012 [61]			RR = 1.15 (0.95–1.40)
**Subject’s SES trajectory **			
Thomson *et al.*, 2004 [58]	(*p* < 0.05)		
**SOCIAL INDEX-Gini**			
Celeste *et al.*, 2009 [53]	OR = 2.49 (2.30–2.68)		
Celeste *et al.*, 2011 [55]	(*p* < 0.05)		

* (+) worse socioeconomic indicator significantly associated to higher severity of dental caries, (−) worse socioeconomic indicator significantly associated to lower severity of dental caries negative association, (#) no significantly association between socioeconomic indicator and dental caries.

Socioeconomic indicators were categorized as follows: schooling of the subject, income of the subject, occupation (of the subject or the subject’s parents, recipient of government benefits), socioeconomic status (of the subject and socioeconomic trajectory) and social index (Gini coefficient) (Table 4). 

In the analysis of the quantitative distribution of the statistical analyses of associations between socioeconomic indicators and dental caries, schooling of the subject was the most frequently used socioeconomic indicator. Lower schooling was statistically associated with greater severity of dental caries in six out of nine multivariate analyses. One study found that lower schooling was associated to lower severity of dental caries, two did not find significant association and one did not find association of schooling of the father and dental caries.

Seven studies analyzed income of the subject and all found that lower income of the subject was significantly associated to greater severity of dental caries. 

Six studies addressed occupation. Two studies found significant association of better occupation of the subject and lower severity of caries; one found the contrary, the better occupation of the subject was significantly associated to higher severity of dental caries. Occupation of the father, mother and recipient of government benefits did not present association with dental caries. 

Lower socioeconomic status of the subject was significantly associated to greater severity of dental caries in two out of two studies. Other two studies did not find significant association. Subject’s socioeconomic trajectory, was significantly associated to dental caries in one study. That means that the low-low and high-low socioeconomic trajectory was significantly associated to more decayed surfaces.

The Gini coefficient was addressed in two studies and they presented that higher scores of Gini coefficient (more vulnerable) was significantly associated with higher severity of dental caries. 

## 4. Discussion and Conclusions

### 4.1. Strengths and Weaknesses of the Review

This systematic review involved the search of multiple electronic databases, with no restrictions regarding language or year of publication. The reference lists of literature reviews were searched for other studies that could also be included. However, it was not possible to search technical reports, papers from research groups or committees and preprints. This could have accounted for some publication bias. One hundred and eighty nine studies were selected for full text analysis in which socioeconomic indicator could have hidden inside the paper as confounder but not as main subject. Efforts were made to try to find studies that reported socioeconomic indicators as risk factors of dental caries.

Due to the permanent nature of socioeconomic indicators, studies that evaluate such indicators tend to be observational rather than interventional. No clinical trials were found although the searches were conducted in three databases related to clinical trials (Controlled-Trial Database, Clinical Trials and the National Institute for Health and Clinical Excellence). 

Most studies (65.85%) were published after the year 2000. Cross-sectional studies were the most common (80.49%). Seven investigations (17.07%) were cohort studies, and one investigation (2.44%) was an ecological study. Among the cohort studies (Table 2), three presented cross-sectional data [62,63,64], one presented data from the start of a prospective longitudinal study [64] and the other presented data on dental caries (DMFT index) at 13 and 20 years of age [62], with only the data at age 20 considered in the analysis. The study of Holst and Schuller [63] presented data from two birth-cohort (1959–1960 and 1929–1938), comprising the ages of 23–24, 45–54 and 35–44 years in 1983; 34–35, 55–64 and 35–44 years in 1994; and 46–47, 68–77 and 35–44 years in 2006. In this study only data from 35–44 years was analyzed. No case-control studies were found. These findings demonstrate a considerable tendency toward conducting and publishing cross-sectional studies and highlight the need for further case-control studies of incident cases, which offer greater scientific evidence through better control of possible methodological biases and data analysis. 

Dental caries is still a health problem in most industrialized countries. In European countries during the XIXth century, rich individuals had more access to sugars and for that reason they had more dental caries. With industrialization there was an increasing provision and consumption of sugar for all populations, not only rich persons. In contrast, it has been observed a decline of dental caries in most industrialized countries over the past 20 years, as a result of a number of public health measures and use of fluorides [65]. However, these measures can be more accessible by individuals of higher socioeconomic status. This can be confirmed by the results, in which six studies observed that subjects with higher income had lower severity of dental caries. In fact, income can give more access to dental services, to fluoridated water, to oral products (toothbrushes and fluoridated toothpastes) and to information about oral health.

### 4.2. Heterogeneity of Studies and Methodological Quality

The diversity of socioeconomic indicators and the parameters used for the cutoff points for dental caries, years of schooling and income demonstrate the heterogeneity of the studies analyzed, thereby meta-analysis was not conducted in this review. The statistical method could render appropriate measure of the strength of the evidence and could assess bias what is a limitation of the review. 

The most frequent socioeconomic indicators were schooling and income (household or *per capita*). There was several cut points used for schooling, which hinders comparisons between studies. Income was generally categorized as high/medium/low, which is somewhat subjective and depends on the definitions of upper, middle and lower class among different countries, as well as on differences in the income limits within each category and the exchange rate between the US dollar and the currency of the country of origin.

In addition to individual data, a small number of studies used collective indicators [53,55,56], such as the Gini coefficient. 

Socioeconomic status was the indicator with the greatest variation in its association with dental caries. The criteria used in each study to classify or group socioeconomic status were variable and subjective. For instance, the Brazilian ABA-Abipeme criteria [32] determine socioeconomic classification by attributing weights to items of domestic comfort and the level of schooling of the head of the family. The socioeconomic classification of the Brazilian population is divided into Classes A, B, C, D and E. One of the limitations of these criteria is the difficulty in comparing the results with findings from international studies because the indicator in question was designed for the Brazilian population. The ABA-Abipeme criteria consider the buying power of the population, which may not be relevant for countries in which access to consumer goods does not adequately portray socioeconomic status. 

There was marked heterogeneity in the criteria used for the evaluation of dental caries, although the majority of studies (68.29%) employed either the mean or median DMFT index and/or its components. The mean or median DMFS was employed in 29.26% of the studies. Other indicators were also used, such as DMFT and DMFS severity. 

The wide variety of population characteristics, age groups and criteria employed in clinical exams hinders the evaluation of the evidence. The large age range of the participants in the studies analyzed (19 to 60 years) may lead to varying results because dental caries are cumulative throughout life. Moreover, the most commonly used indices in the studies (DMFT and DMFS) consider both the past and present history of dental caries. The age group proposed by the WHO for studies on adults (35 to 44 years) was used in only 15 studies.

### 4.3. Statement of Principal Findings

The strength of the evidence included in the present review was affected by a number of methodological issues. Despite the heterogeneity of the socioeconomic indicators, there was scientific evidence of associations between dental caries and the subjects’ schooling, income, and occupation, as well as the Gini coefficient. A lower level of schooling was associated with more dental caries in the statistical analyses that addressed the subjects’ schooling. The subjects’ occupation was associated with lower severity of dental caries, whereas the parents’ occupation presented no association with dental caries. These findings were expected from the adult population because the influence of one’s mother and father is more appropriately evaluated in studies involving children or adolescents. Subjects’ income was also associated with dental caries, although the criteria for the assessment of income differed among the different studies (in terms of currency in each country and the exchange rate used for the conversion into dollars).

Socioeconomic status demonstrated considerable variability in the results of the association with dental caries. It is possible that the classifications used for this variable affected this finding. Although socioeconomic status is generally classified as high, medium, or low, this subjective classification depends on the researcher’s assessment. 

Among the studies that employed the Gini coefficient, two analyses were statistically associated to dental caries. The analysis of economic inequality between countries is mainly based on the interpretation of this coefficient, which is widely used in the literature because it reveals the degree of inequality in the distribution of income in a specific setting [66]. The Gini index considers information regarding the mean income of heads of families and compares the proportion of the total income of a portion of the population in relation to the weight of this subpopulation in the general population [67]. Therefore, the Gini coefficient evaluates the concentration of income without considering the social factor of schooling. In other words, it only evaluates the economic determinant. This index should therefore be used together with other indices to assess social determinants. For example, the Human Development Index is an international indicator that considers education, income and longevity. This index can be used as a complement to the Gini index because it also has limitations and has been criticized for its inadequate treatment of income, lack of comparability between survey years and different assessments of development between groups of countries [68]. 

Only 21 studies out of 41 studies employed multivariate analyses, and the remaining studies failed to adjust for confounding variables. The use of bivariate analysis alone can result in biased results regarding associations between socioeconomic indicators and dental caries. These studies are described in Table 1, Table 2, Table 3 and highlighted the importance of the adjustment for confounding variables in studies. Multivariate analysis becomes important since the theoretical models of social determinants of health [69], and theoretical models of the determinants of dental caries [2,9] have measured social determinants of health at different levels of society [7].

The quality of the studies ranged between five and nine points, which demonstrates methodological variability. Most of the investigations were cross-sectional studies. This type of design offers a lower degree of scientific evidence compared with case-control and cohort studies. Regarding the Newcastle-Ottawa quality assessment scale, lower scores were mainly related to comparability (lack of a multivariate analysis), non-response rate and ascertainment of exposure (non-blinded interview). However, the use of scales for quality assessment has limitations that should be considered. The scales use a summary score that involves weights to different items and it is difficult to justify the weights assigned. Some authors have considered them of unreliable validity and less transparent to the users of the reviews [70]. Besides that, the use of the scale and weight criteria can be very subjective among reviewers. On the other hand, the Newcastle-Ottawa scale has been used to assess quality and it is able to standardize the scores among all studies. Newcastle-Ottawa scale was used for scoring studies, but methodological analysis of the studies was not based on it. Instead it was used narrative approach that could be fully reported by the present review. 

The lack of participants in the oral exam (*i.e.*, a lower number of individuals examined in relation to the total number of participants) was another negative aspect. Only nine studies (21.95%) did not experience this loss. The non-participation of individuals in exams can result in data that do not adequately portray the population because there is no way of knowing whether those who refused to undergo the exam have a better or worse oral health status. The final important issue is the lack of external validity. Many of the studies offered no information regarding the sample size calculation and/or the analyzed population constituted a convenience sample.

### 4.4. Suggestions for Further Research

There is evidence of the association between socioeconomic indicators and dental caries in adults. However, the magnitude of evidence needs to be further evaluated. The strength of evidence could be hard to evaluate because it needed to know if a study adjusted the association for a true confounding factor or for mediators. There is a need for theoretical model to identify the role of each variable and it should be very clear throughout the study.

Studies that evaluate dental caries in subjects with wide range of age should perform adjusted analysis as odds ratios for controlling the age. The measurement of dental caries and socioeconomic indicators should be performed by different researchers to avoid or minimize the influence of these indicators on responses and on possibly biasing the results.

In conclusion, the findings of the present systematic review provide evidence that worse socioeconomic indicators, such as subject’s schooling, income, occupation and the Gini coefficient, are associated with a greater severity of dental caries in adults. There was considerable degree of heterogeneity in the methodology, socioeconomic indicators and classification of dental caries was found in the studies analyzed. More cohort and case-control studies of incidence cases are needed to establish the magnitude of the scientific evidence regarding the association between socioeconomic indicators and dental caries. 
